# Dynamic Compressive and Flexural Behaviour of Re-Entrant Auxetics: A Numerical Study

**DOI:** 10.3390/ma16155219

**Published:** 2023-07-25

**Authors:** Dianwei Gao, Jianhua Zhang, Chunwei Zhang, Yun You

**Affiliations:** 1 School of Architecture and Civil Engineering, Shenyang University of Technology, Shenyang 110870, China; gaodianwei@sut.edu.cn; 2 Multidisciplinary Center for Infrastructure Engineering, Shenyang University of Technology, Shenyang 110870, China; zhangchunwei@sut.edu.cn; 3 Liaoning Provincial Key Laboratory of Infrastructure Engineering Safety and Protection, Shenyang University of Technology, Shenyang 110870, China; 4 College of Aerospace and Civil Engineering, Harbin Engineering University, Harbin 150001, China; 5 Shanghai Merchant Ship Design & Research Institute, Shanghai 201203, China; youyun0922@126.com

**Keywords:** finite element modelling, negative Poisson’s ratio, auxetic structures, metamaterials, dynamic properties

## Abstract

Re-entrant auxetics offer the potential to address lightweight challenges while exhibiting superior impact resistance, energy absorption capacity, and a synclastic curvature deformation mechanism for a wide range of engineering applications. This paper presents a systematic numerical study on the compressive and flexural behaviour of re-entrant honeycomb and 3D re-entrant lattice using the finite element method implemented with ABAQUS/Explicit, in comparison with that of regular hexagonal honeycomb. The finite element model was validated with experimental data obtained from the literature, followed by a mesh size sensitivity analysis performed to determine the optimal element size. A series of simulations was then conducted to investigate the failure mechanisms and effects of different factors including strain rate, relative density, unit cell number, and material property on the dynamic response of re-entrant auxetics subjected to axial and flexural loading. The simulation results indicate that 3D re-entrant lattice is superior to hexagonal honeycomb and re-entrant honeycomb in energy dissipation, which is insensitive to unit cell number. Replacing re-entrant honeycomb with 3D re-entrant lattice leads to an 884% increase in plastic energy dissipation and a 694% rise in initial peak stress. Under flexural loading, the re-entrant honeycomb shows a small flexural modulus, but maintains the elastic deformation regime over a large range of strain. In all cases, the compressive and flexural dynamic response of re-entrant auxetics exhibits a strong dependence on strain rate, relative density, and material property. This study provides intuitive insight into the compressive and flexural performance of re-entrant auxetics, which can facilitate the optimal design of auxetic composites.

## 1. Introduction

Counterintuitively, auxetic structures contract or expand transversely under uniaxial compressive or tensile loads, respectively. Naturally occurring auxetic structures can be identified in crystalline materials, cancellous bones, animal skins, and the cytoskeleton of red blood, while man-made auxetic structures were first introduced by Gibson et al. [1] in 1982 in the form of 2D re-entrant honeycombs. There has been an increasing amount of scientific interest and a rise in the number of applications of auxetic structures in practical engineering since the 3D auxetic foams were manufactured by Lakes [2]. Typically, auxetic structures under compressive loading can exhibit superior indentation resistance, fracture resistance, energy absorption capacity, and volume compressibility, etc., and thus have great potential in a wide range of applications such as angioplasty stents, foldable batteries, anti-pulling nails, and anti-impact/blast armours [3]. Recently, polymer auxetic structures were combined with cement for the construction of ductile building components in civil engineering. Therefore, as promising metamaterials used in a variety of engineering works, the mechanical behaviour of auxetic structures under both compression and flexure should be studied comprehensively.

In the past thirty years, a large number of theoretical, experimental, and numerical studies have been conducted to evaluate the mechanical performance of auxetic structures given their advanced deformation characteristics. So far, the compressive behaviour of auxetic structures under compressive loading has been extensively studied, while the flexural behaviour of them under transverse impact has been rarely explored. Moreover, the guidance on how to choose the optimal auxetic structures in engineering design is still limited. Thus, a direct comparison of the basic compressive and flexural mechanical properties of various auxetic structures accounting for the same impact scenarios is of importance for the optimal design of auxetics. To date, the main research objectives of auxetic structures in the published literature can be classified into two categories, namely, pure auxetic structures and auxetic composite structures.

Regarding pure auxetic structures, the existing studies are mainly focused on the static and dynamic mechanical behaviour of re-entrant auxetic structures under compressive loading because of the simple structural forms and relatively intuitive deformation mechanisms. Given the unit cell geometries, re-entrant auxetic structures can be categorised into three types: 2D re-entrant honeycombs, 3D re-entrant lattices, and 3D re-entrant foams. A series of numerical studies has been carried out to estimate the effects of geometric parameters and impact scenarios on the in-plane compressive behaviour of 2D re-entrant auxetic structures. Mimicking plane strain behaviour, the effects of microstructural imperfections (e.g., irregularity and residual convex units) and unit cell geometry (e.g., cell-wall angle and shape ratio) on compressive mechanical properties were numerically estimated [4,5]. For comparison, Liu et al. [6] predicted the in-plane dynamic compressive mechanism, impact force, and energy absorption capacity of both conventional hexagonal honeycomb and re-entrant honeycomb by finite element simulations. Rahul et al. investigated the in-plane behaviour and failure modes of re-entrant auxetic structures under inclined compression. The effect of loading angle on the transition stages and stress–strain curves has been discussed [7,8].

Based on previous studies, some attempts were made to enhance the in-plane auxetic behaviour and mechanical properties such as the stiffness, compressive strength, and energy absorption capacity of 2D re-entrant honeycombs. Khan et al. [9] introduced an extra horizontal member between the vertical and inclined members of 2D re-entrant honeycomb to enhance the directional properties such as the negative Poisson’s ratio effect. The novel design with embedded ribs was proposed to enhance the in-plane Young’s modulus and critical buckling strength of 2D re-entrant honeycomb [10,11]. Ingrole et al. [12] carried out a comparative study of regular hexagonal honeycomb, 2D re-entrant honeycomb, locally reinforced auxetic-strut structure, and hybrid structure composed of regular hexagonal honeycomb and auxetic-strut structure in terms of static compressive behaviour and energy absorption. Test specimens were fabricated using a 3D-printing method with an acrylonitrile butadiene styrene (ABS) filament. Based on the same procedure, Wu et al. [13] evaluated the in-plane impact resistance and energy absorption capacity of cell-wall graded 2D re-entrant honeycomb and a uniform one, considering the effects of impact velocity, cell-wall angle gradient, and cell-wall thickness. Three-dimensional re-entrant lattices are of importance to provide the concept and possibility for man-made 3D auxetic structures. With 3D printing, the manufacturing process of 3D re-entrant lattice becomes relatively simple, which facilitates the experimental tests [14,15,16,17,18,19].

Apart from re-entrant auxetic structures, chiral honeycomb became popular after it was first proposed by Prall and Lakes [20] in 1997. The chiral honeycomb with given geometric confinement was found to have an in-plane negative Poisson’s ratio of −1 from theoretical analysis and experimental testing; existing studies are mainly focused on the elasto-static mechanical properties [21,22,23,24], while the dynamic plastic behaviour of chiral honeycomb has not been extensively addressed [25,26]. Although pure auxetic structures can provide superior properties as lightweight porous structures, one of the main challenges is how to improve their capability in resisting heavy external loads and, thus, auxetic composite structures were proposed.

With respect to auxetic composite structures, to date, a few studies have investigated the blast or impact resistance of sandwich structures and metal tubes with auxetic cores. The frequently used auxetic cores are 2D re-entrant honeycomb [27] and 3D re-entrant lattice [28]. Adopting a graded core configuration, the combination of auxetic structure and conventional hexagonal honeycomb was employed to undergo impact loading [29]. Composite chiral honeycomb has a unique ability to offer large deformation, while the shape remains invariable and can thus be used for morphing airfoils [30]. In recent years, some experimental and numerical studies on auxetic structures–concrete composite structures have been undertaken, indicating that the interlock mechanism of auxetic honeycomb and concrete can effectively enhance the ductility of concrete in compression and bending [31,32].

Although the behaviour of auxetic composites with several types of auxetic core structures under compressive and flexural loadings has been studied, the emphasis has been mainly placed on the overall performance for whole structures. However, the lack of a direct comparison of mechanical performance of the commonly used auxetic core structures (e.g., 2D re-entrant honeycomb and 3D re-entrant lattice) would limit the development and widespread application of them and auxetic composite structures consisting of them. In addition, the axial compressive behaviour of auxetic core structures has been extensively explored, while the behaviour of auxetic core structures under flexural loading has not been fully understood. The main purpose of this study is to systematically investigate the compressive and flexural behaviour of re-entrant honeycomb (R-H) and 3D re-entrant lattice (3D R-L) using the finite element (FE) method. As one of the most widely used lightweight structures, a regular hexagonal honeycomb (H-H) is used as a reference structure for comparison. FE models were developed with ABAQUS 6.14-4 and validated against the literature experimental data in terms of stress–strain relationships. Afterwards, the mesh size sensitivity analysis was conducted to determine the element size for the FE model, which was then adopted to simulate the behaviour of different auxetic structures under compressive and flexural loading in terms of failure mode, stress–strain/load-deflection response, and energy dissipation with a special focus on the effects of strain rate, relative density, unit cell number, and material property. Based on the simulations, the underlying mechanisms of the dynamic compressive and flexural behaviour of re-entrant auxetics are discussed in depth. The obtained results could offer insights into the construction of composite structures using auxetic cores, e.g., developing ductile building components in civil engineering with the combination of polymer auxetic structures and cement.

## 2. Finite Element Modelling

To effectively simulate the failure modes and mechanical performance of auxetic structures under compressive and flexural loading, the FE model based on ABAQUS/Explicit was developed and validated before its application, which is presented in detail below.

### 2.1. Geometry

**Figure 1:**
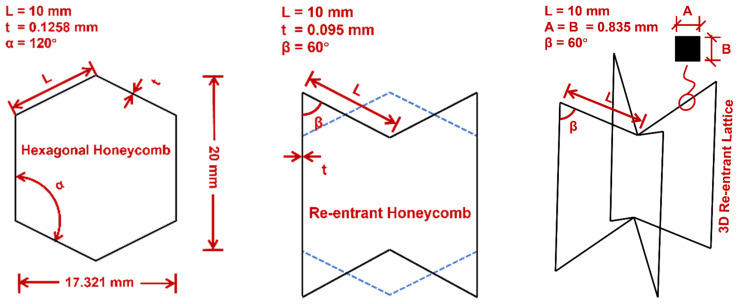
Unit cell geometry of reference structure (hexagonal honeycomb: H-H) and auxetic structures (re-entrant honeycomb: R-H; 3D re-entrant lattice: 3D R-L).

In this study, two auxetic structures including re-entrant honeycomb (R-H) and 3D re-entrant lattice (3D R-L) were considered for comparative study and a hexagonal honeycomb (H-H) was adopted as the reference structure for comparison. To provide consistency for comparison across different types of auxetic structures, all unit cells kept the same initial in-plane dimension of approximately 17.32 mm in width and 20 mm in height. The relevant unit cell geometry and key parameters for each auxetic structure are shown in Figure 1. Under such an initial geometry condition, all of the specimens can ensure the same relative density, i.e., ρ* = 1.5%. The relative density is defined as *ρ*_s_/*ρ*_c_, where *ρ*_s_ the equivalent density of the specimen, while *ρ*_c_ is the density of the constituent material. The equivalent density *ρ*_s_ is calculated using the mass and volume of the specimen.

**Figure 2:**
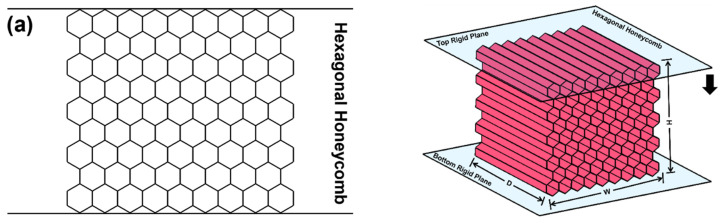

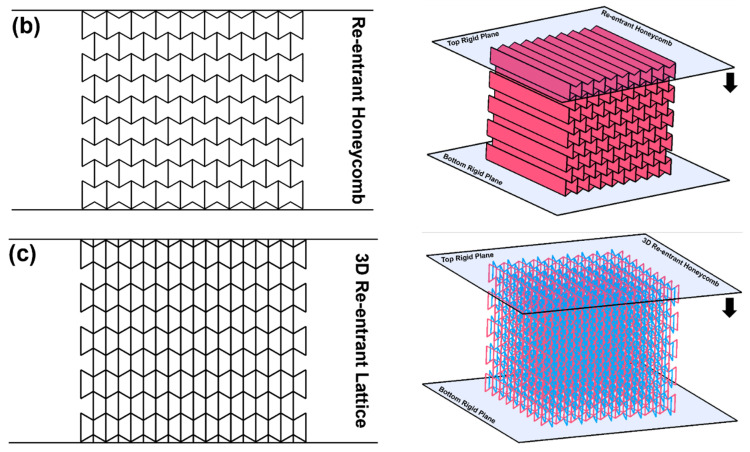
Specimens for compressive simulations: (**a**) hexagonal honeycomb, (**b**) re-entrant honeycomb, and (**c**) 3D re-entrant lattice.

Figure 2 illustrates the specimen details for FE simulations of compression. Each specimen consisted of nine unit cells in both width and height directions. The specimens for the regular hexagonal honeycomb and 2D re-entrant honeycomb were constructed from their 2D geometries by extruding in the out-of-plane direction. The 3D re-entrant lattice was assumed to have a square section. The compressive simulations were conducted on rectangular specimens with dimensions of 155.88 mm × 140 mm × 155.88 mm (W × H × D). Each specimen was sandwiched in between two rigid walls for the fixture.

**Figure 3:**
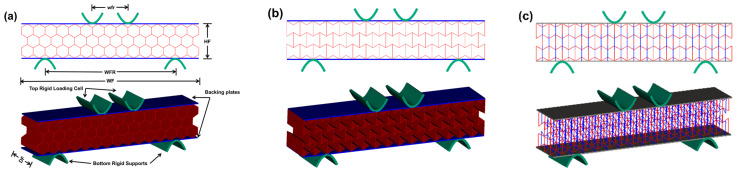
Specimens for flexural simulations: (**a**) hexagonal honeycomb, (**b**) re-entrant honeycomb, and (**c**) 3D re-entrant lattice.

Figure 3 shows the details of the specimens for flexural simulations. Each specimen was composed of 15 and 3 unit cells in the directions of width and height, respectively. The flexural simulations were conducted on rectangular specimens with a dimension of 260 mm × 50 mm × 50 mm (WF × HF × DF). Each specimen was sandwiched in between two backing plates to prevent the premature local failure of the material near the loading cells. There is no glued connection between the rigid plates and the specimen. The value of thickness of the backing plates for all of the considered samples is set to 0.5 mm. The four-point bending tests were simulated with a support span of WFR and a load span of wrf, as illustrated in Figure 3a.

### 2.2. Material Properties

The elastic perfectly plastic material constitutive model was applied to emulate the aluminium alloy-based reference structure and auxetic structures, which has been proven to be reasonable for the simulations of the impact behaviour of thin-wall aluminium structures such as hexagonal honeycombs [33], arrowhead honeycombs [34], and re-entrant honeycombs [6]. Although the deformation of honeycomb structures is sensitive to impact velocity, aluminium alloys have no obvious strain rate effect under impact loading. Acrylonitrile butadiene styrene (ABS) polymer, which is an important plastic material for 3D printing, was also selected as the material for the auxetic structures. Table 1 gives the details of the material properties. It should be noted that the elastic perfectly plastic material constitutive model has been proven to be reasonable for simulations of the impact behaviour of thin-wall aluminium structures [6,25,33,34]. Therefore, in the present study, the ABS material model was used for validation.

### 2.3. Finite Element Model

#### 2.3.1. Element Type and Mesh Size

Both the honeycomb structures and rigid walls were modelled using shell elements of S4R with reduced integration and a default hourglass control algorithm. An element size of 2.5 mm was adopted for FE meshing of all of the honeycomb specimens, leading to a maximum of 1,079,998 elements and 203,400 elements for compressive and flexural simulations, respectively. The 3D re-entrant lattices were modelled using three-node linear Timoshenko beam elements (B31), allowing the transverse shear deformation of the beam. The element length of 3D re-entrant lattices was 0.5 mm, and the total element number for compressive and flexural simulations was 149,600 and 30,400, respectively. The rigid walls and backing plates had an element size of 5 mm.

#### 2.3.2. Load and Boundary Conditions

For the compressive simulation, the bottom rigid plane was fixed at all six degrees of freedom (DoFs) of the reference node. The top rigid plane was constrained at the reference node that was only allowed to move downwards. All of the DOFs for the FE model of the specimen were activated. To reduce the penetration, the initial distance between the specimen and the top rigid plane and the bottom rigid plane was assigned to be half of the wall thickness. Similarly, for the flexural simulations, the bottom rigid supports were fixed at all DOFs of the reference nodes. The top rigid loading cells could only travel downwards. No constraints were applied to the specimen and backing plates.

#### 2.3.3. Solving Procedure

Abaqus/Explicit provides two algorithms for simulating the contact interactions among different parts for FE simulations, i.e., the general contact algorithm and the contact pair algorithm [25]. The general contact algorithm is the default self-contact algorithm in ABAQUS/Explicit, which was adopted in this study to automatically include all of the possible contact interactions during the impact processes. In this algorithm, the penalty contact formulation was enforced to introduce contact constraints between the surfaces, and the penalty stiffness enforcing a “hard” contact option was chosen automatically by the code. The Coulomb friction model was employed to consider the frictional forces between contacting surfaces with a friction coefficient of 0.2.

### 2.4. Validation of Finite Element Model

#### 2.4.1. Mesh Size Sensitivity

**Figure 4:**
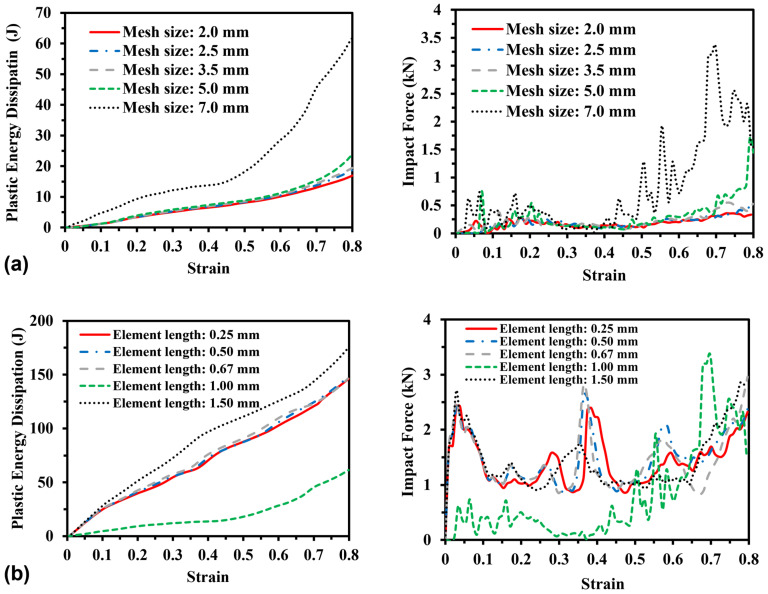
Mesh size sensitivity of plastic energy dissipation and impact stress–strain curves (*V* = 5 m/s) for (**a**) re-entrant honeycombs and (**b**) 3D re-entrant lattices.

To obtain a proper mesh size for the FE model with the minimum number of elements, the sensitivity analysis of mesh sizes of 2.0, 2.5, 3.5, 5.0, and 7.0 mm on simulation results in terms of plastic energy dissipation and impact stress–strain relationship was conducted and is presented in Figure 4. As seen in Figure 4a,b, the simulation results corresponding to the mesh size of 2.5 mm are sufficiently convergent and adaptable; thus, it was adopted as the mesh size for the simulations below.

#### 2.4.2. Comparison with Experimental Results

**Figure 5:**
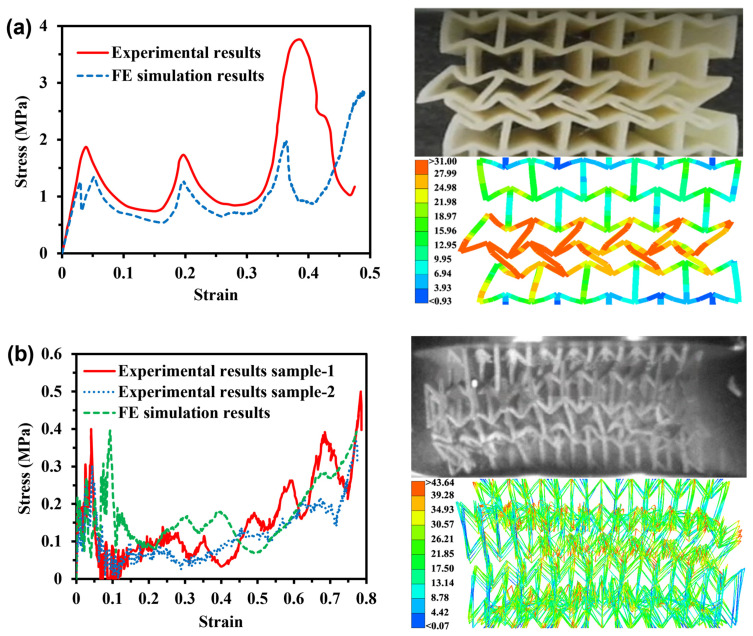
Comparison of simulation and experimental results [12,14] in terms of impact stress–strain curves and deformation shapes for (**a**) re-entrant honeycomb and (**b**) 3D re-entrant lattice.

Herein, the capability of FE models to capture the failure modes and stress–strain response of auxetic structures is validated with the available experimental tests in the literature for re-entrant honeycomb [12] and 3D re-entrant lattice [14]. In the quasi-static compressive test for the re-entrant honeycomb, the sample had dimensions of 45 × 45 × 40 mm^3^ (length × width × height). In the dynamic compressive test for the 3D re-entrant lattice, the sample had dimensions of 65 × 65 × 56 mm^3^, and a compressive velocity of 5 m/s. Figure 5 indicates a good agreement between the simulations and experimental data. However, minor discrepancies between the simulated and experimental results can be observed in Figure 5a,b. This may be partially attributed to the discrepancy between the 3D-printed specimens and the numerical models. However, the simulated average stress, 1.03 MPa in Figure 5a and 0.165 MPa in Figure 5b are very similar to the average experimentally measured average stress 1.08 MPa in Figure 5a and 0.166 Mpa in Figure 5b, with a difference of only 4.6% and 0.6%, respectively, and the trend is similar.

## 3. Compressive Behaviour of Re-Entrant Auxetics

This section presents the simulation results of the dynamic compressive behaviour of 2D re-entrant honeycombs and 3D re-entrant lattices in comparison with that of regular hexagonal honeycombs, in terms of failure modes, stress–strain curves, and energy dissipation. Emphasis is placed on the effects of four critical parameters including strain rate (compressive speed), relative density, unit cell number, and material property. All calculations were conducted up to the strain of 0.8. Three different values of impact speed, i.e., 5 m/s, 15 m/s, and 25 m/s, were considered, corresponding to the strain rates varying from 35.71 to 178.57 s^−1^. The velocity range was determined by referring to a normalised velocity $\bar{V}=V/(c_{0}\varepsilon_{Y})$, where $c_{0}={\left(E/\rho \right)}^{0.5}$ represents the elastic wave speed in the constituent material and $\varepsilon_{Y}=\sigma_{Y}/E$ [35]. In this study, $\bar{V}$ is in the range of 0.53–2.64, covering both the low-speed impact ($\bar{V}<1$) and high-speed impact ($\bar{V}>1$).

### 3.1. Failure Modes

**Figure 6:**
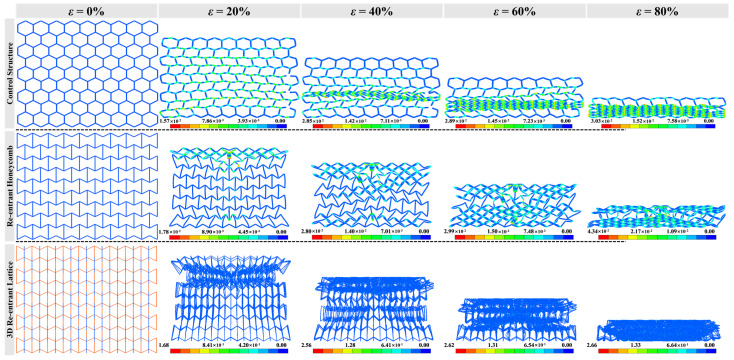
Deformed shapes with crushing strain of H-H, R-H, and 3D R-L at compressive speed of *V* = 5 m/s while the total mass of structures remains constant at 0.14 kg.

**Figure 7:**
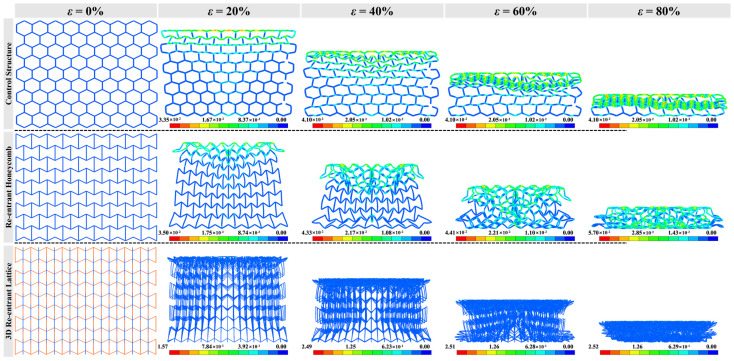
Deformed shapes with crushing strain of H-H, R-H, and 3D R-L at compressive speed of *V* = 15 m/s while the total mass of structures remains constant at 0.14 kg.

**Figure 8:**
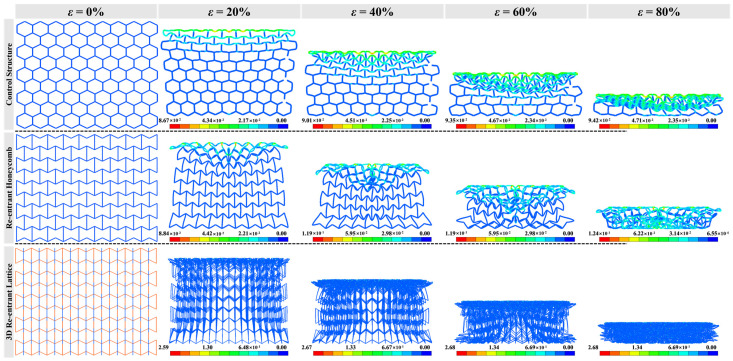
Deformed shapes with crushing strain of H-H, R-H, and 3D R-L at compressive speed of *V* = 25 m/s while the total mass of structures remains constant at 0.14 kg.

Figure 6, Figure 7 and Figure 8 show the compressive deformation of reference structure and re-entrant auxetics at various compressive strains. The global deformation can be observed in all cases before failure. Apart from the reference structure (H-Hs) that extends transversely, R-Hs and 3D R-Ls experience shrinkage towards the crossover due to a negative Poisson’s ratio effect. When it is subjected to a low-speed compression (e.g., *V* = 5 m/s), the local buckling failure takes place obviously from the middle of H-H, at both ends of R-H, and the proximal end of 3D R-L, respectively. As the compressive speed increases to 15 m/s, the densification of cell walls and cell ribs almost starts at the proximal ends and grows with the motion of the top rigid plane, which can be attributed to the increased dynamic effect. The growth of major densification for H-H and 3D R-L forms row by row, but in a diamond shape for R-H. With the increase in compressive speed to 25 m/s, the failure mode changes slightly, with a closer densification region to the proximal ends than that under the compressive speed of 15 m/s.

On top of the compressive speed, the effects of relative density, unit cell number, and material property on the failure modes of reference structures and re-entrant auxetics are also investigated, the results of which are listed in Table 2. It can be observed that the failure modes are sensitive to compressive speed, except for 3D R-Ls, which almost remain in dynamic failure mode. Generally, the compressive failure modes are slightly influenced by relative density and unit cell number for the present models.

### 3.2. Stress–Strain Curves

**Figure 9:**
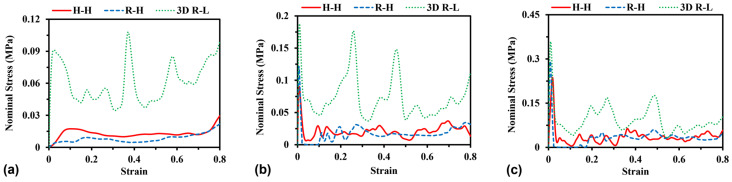
Stress–strain curves of H-H, R-H, and 3D R-L at a compressive speed of (**a**) *V* = 5, (**b**) 15, and (**c**) 25 m/s, respectively.

Corresponding to different failure modes, Figure 9 displays the nominal stress–strain curves of different models with respect to the compressive speeds of 5, 15, and 25 m/s, respectively. In the present study, the compressive force and cross-sectional area of the model are used to calculate the nominal stress. The stress–strain curve for each model experiences a steady increase in stress up to the peak stress, followed by the so-called plateau stress, although it presents a wave feature. The compressive stress levels of H-H and R-H are relatively close, but much lower than that of 3D R-L, which can be explained by the higher failure stress of micro-structural cells and more unit cells of 3D R-L than those of H-H and R-H.

#### 3.2.1. Effect of Strain Rate

**Figure 10:**
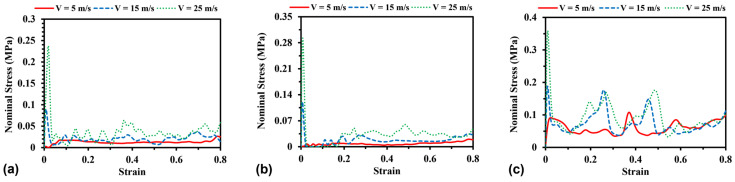
Stress–strain curves of (**a**) H-Hs, (**b**) R-Hs, and (**c**) 3D R-Ls at various values of compressive speed while the total mass of models remains constant at 0.14 kg.

The effect of strain rates on the compressive response of different models was estimated by changing the compressive speed between 5 m/s and 25 m/s, while the total mass of the models maintained a constant of 0.14 kg. Figure 10 illustrates the stress–strain curves for different models against compressive velocity. As expected, increasing the compressive strain rate can always increase the stress levels for all models in the present study due to the strain rate effect of porous structures. For instance, the average stress calculated between strains of 0.1 and 0.6 among the different models increases with the increase in compressive speed. The average stress of R-H shows the most sensitivity to compressive speed. Specifically, there is a 148%, 403%, and 83% increase for H-H, R-H, and 3D R-L, respectively, when increasing the compressive speed from 5 m/s to 25 m/s. In addition, the rise in the strain rate causes a more severe fluctuation of the stress–strain curve, which can be ascribed to the more significant dynamic effect of changing cell walls or ligaments from bending failure to combined axial and shear failure.

#### 3.2.2. Effect of Relative Density

**Figure 11:**
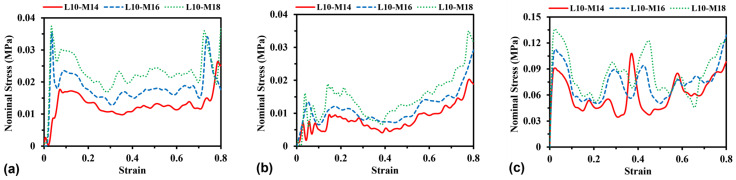
Stress–strain curves of (**a**) H-Hs, (**b**) R-Hs, and (**c**) 3D R-Ls with various values of relative density while the compressive speed remains constant at 5 m/s.

To compare the results of different models on the same ground, a parametric study was conducted to investigate the effect of relative density on the stress–strain curves by changing the wall thickness for H-Hs and R-Hs and the cross-sectional area of beams for 3D R-Ls. Accordingly, three values of the total model mass of 0.14 kg, 0.16 kg, and 0.18 kg, respectively corresponding to values of model relative density of approximately 1.52%, 1.75%, and 1.98%, were considered. Figure 11 shows the variations of the stress–strain curves with model relative density for H-Hs, R-Hs, and 3D R-Ls, indicating that the stress–strain curves are dependent on the relative density. The models with thicker walls or larger cross-section areas exhibit larger compressive stress the corresponding larger buckling loads. However, the change in relative density has less effect on the characteristics of the stress–strain curves, which can be explained by the relatively similar failure modes of the present models under given loading conditions. In other words, the change in relative density has a minor effect on the deformation process of different models in the present study. This finding is in good agreement with the experimental results reported for regular hexagonal honeycomb and auxetic honeycomb [5,6]. In addition, with the change in relative density from 1.52% to 1.98%, the average compressive load rises by 77.4%, 79.8%, and 48.7% for H-Hs, R-Hs, and 3D R-Ls, respectively. The compressive stress of 3D R-L shows relatively lower sensitivity to structural relative density compared to that of honeycomb structures, which have similar increase rates.

#### 3.2.3. Effect of Unit Cell Number

**Figure 12:**
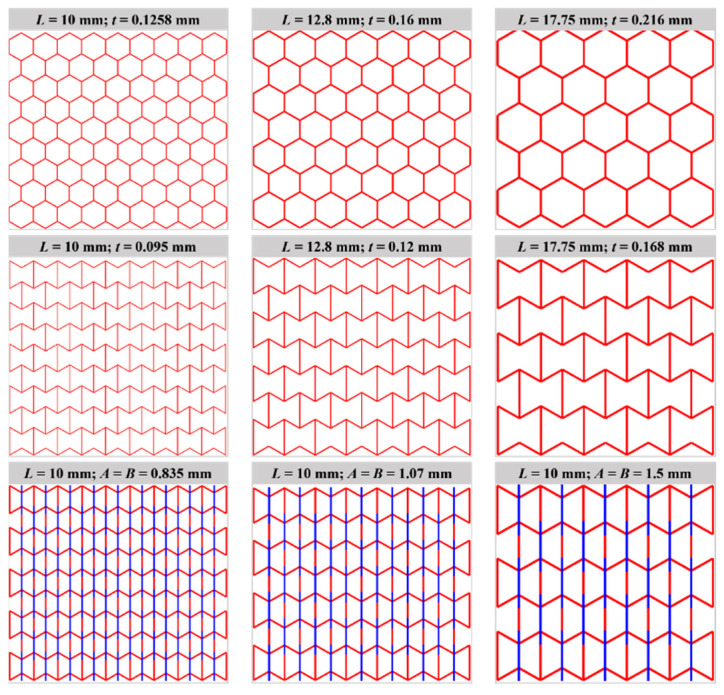
Geometry of compressive models when keeping total dimension and mass constant.

**Figure 13:**
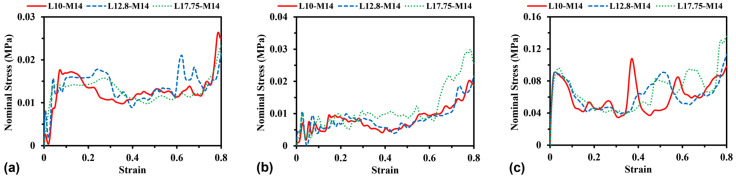
Stress–strain curves of (**a**) H-Hs, (**b**) R-Hs, and (**c**) 3D R-Ls corresponding to various unit cell numbers while the total mass remains constant at 0.14 kg.

To investigate the effect of unit cell number on the stress–strain curves, simulations were performed by changing the side length *L* between 10 mm and 17.75 mm (e.g., see Figure 12) while keeping the dimension and mass unchanged for all models. Herein, the total mass of the models keeps a constant of 0.14 kg by varying the wall thickness for H-Hs and R-Hs and the cross-sectional area of beams for 3D R-Ls. Figure 13 shows the curves of nominal stress–compressive strain of different models corresponding to in-plane unit cell numbers varying from 9 × 9 to 5 × 5 (W × H). It can be observed that changing the unit cell numbers does not significantly influence the characteristics of the stress–strain curves compared to the change in strain rate and relative density of the models, since these cases correspond to the same failure modes shown in Table 2.

#### 3.2.4. Effect of Material Property

**Figure 14:**
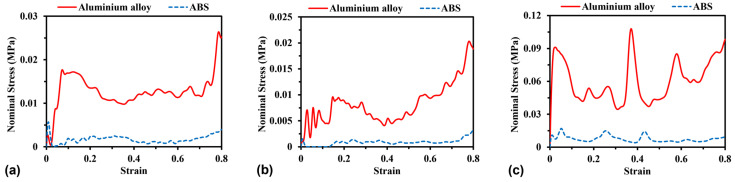
Stress–strain curves of (**a**) H-Hs, (**b**) R-Hs, and (**c**) 3D R-Ls corresponding to different material properties while the compressive speed remains constant at 5 m/s.

Aluminium alloy and ABS (acrylonitrile butadiene styrene) are the most commonly used base materials to manufacture auxetic structures. In order to evaluate the effect of material properties on the stress–strain relationship, simulations were conducted for the various models made of both aluminium alloy material properties and ABS material properties. The material behaviour of aluminium alloy and ABS were simulated using the elastic perfectly plastic material constitutive model. As seen in Figure 14, as expected, the aluminium alloy models show significantly higher compressive stress than the ABS models, since the aluminium alloy models possess higher elastic modulus and yield strength than that of ABS models. The replacement of ABS models by aluminium alloy models leads to an increase in the average compressive stress of H-Hs, R-Hs, and 3D R-Ls by 703%, 710%, and 629%, respectively.

### 3.3. Energy Dissipation

**Figure 15:**
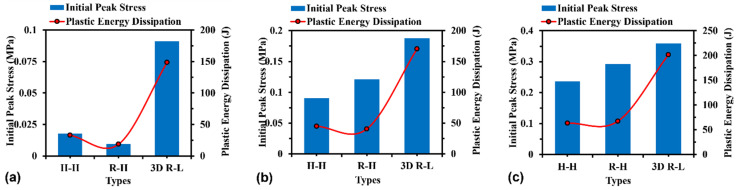
Initial peak stress and plastic energy dissipation of H-H, R-H, and 3D R-Ls at a compressive speed of (**a**) *V* = 5, (**b**) 15, and (**c**) 25 m/s, respectively.

Plastic energy dissipation capacity and initial peak stress are two important parameters for evaluating structural performance in terms of compressive resistance. It is well known that when subjected to compressive loading, the protective structures present relatively low initial peak stress, while high plastic energy dissipation is desired in practical engineering. Figure 15 compares the simulated results in terms of the initial peak stress and plastic energy dissipation for H-Hs, R-Hs, and 3D R-Ls at the compressive speeds of 5, 15, and 25 m/s, respectively. The initial peak stress represents the maximum value of the nominal compressive stress before model failure. Three-dimensional R-Ls exhibit superior plastic energy dissipation capacity over H-Hs and R-Hs, but the highest initial peak stress regardless of compressive speed. Especially for low-speed (*V* = 5 m/s) compression, the 3D re-entrant lattice can resist 7.94 times more compressive stress and dissipate 9.84 times more plastic energy than the re-entrant honeycomb. In addition, under low-speed compressive loading, H-H shows both higher initial peak stress and plastic energy dissipation than R-H. This suggests that the hexagonal honeycomb has greater capacity in impact resistance and plastic energy dissipation than the re-entrant honeycomb. Specifically, the hexagonal honeycomb resists 1.76 times as much compressive stress and dissipates 1.87 times as much plastic energy as the re-entrant honeycomb. Nevertheless, for both H-Hs and R-Hs, although the initial peak stress and plastic energy dissipation increase with increasing compressive speed, R-Hs show a higher potential in terms of impact resistance and plastic energy dissipation at high-speed impact. Specifically, the re-entrant honeycomb resists 1.23 times the compressive stress and dissipates 1.06 times the plastic energy compared with the hexagonal honeycomb when the compressive speed rises to 25 m/s. This can be attributed to the stronger NPR effect of R-Hs at high-speed compressive loading than that at low-speed compressive loading (see Figure 6, Figure 7 and Figure 8).

#### 3.3.1. Effect of Strain Rate

**Figure 16:**
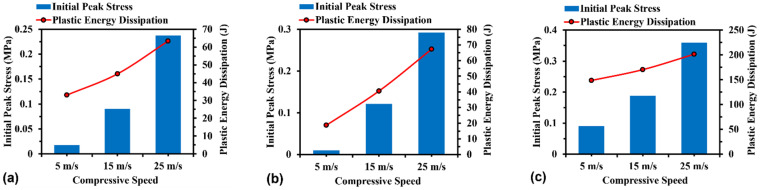
Initial peak stress and plastic energy dissipation of (**a**) H-Hs, (**b**) R-Hs, and (**c**) 3D R-Ls at various values of compressive speed while the total mass of structures remains constant at 0.14 kg.

Figure 16 displays the compressive initial peak stress and plastic energy dissipation at compressive speeds varying from 5 m/s to 25 m/s. It can be observed that both the initial peak stress and plastic energy dissipation have a positive correlation with strain rate for all models. Relatively, the compressive response of H-Hs and R-Hs is more sensitive to compressive strain rates than that of 3D R-Ls due to the convoluted stress wave propagation paths through 3D R-Ls. As expressed below, the plastic energy dissipation increases exponentially with the increase in compressive velocity [36]:

(1) $$PE=\int \sigma_{i}Ads$$

(2) $$\sigma_{i}={\left(\sigma_{ss}+\frac{\rho V^{2}}{\varepsilon_{d}}\right)}_{i}$$

where *PE* is the plastic energy dissipation, $\sigma_{i}$ is the dynamic plateau stress of regular hexagonal honeycomb, $\sigma_{ss}$ is the static plateau stress, ρ is the density of structure, *V* is the compressive velocity, and $\varepsilon_{d}$ is the locking strain of the structure under static loading.

#### 3.3.2. Effect of Relative Density

**Figure 17:**
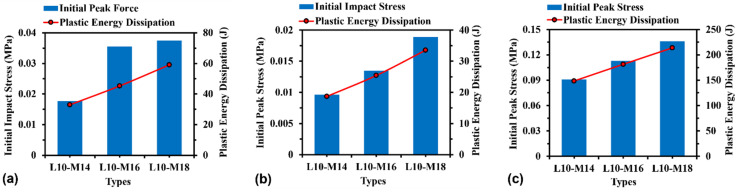
Initial peak stress and plastic energy dissipation of (**a**) H-Hs, (**b**) R-Hs, and (**c**) 3D R-Ls with various values of relative density while the compressive speed remains constant at 5 m/s.

The effect of relative density on initial peak stress and plastic energy dissipation can be assessed by changing the wall thickness for H-Hs and R-Hs, as well as changing the cross-sectional area of beams for 3D R-Ls, which results in the change of model mass accordingly between 0.14 kg and 0.18 kg. Figure 17 shows the initial peak stress and plastic energy dissipation for H-Hs, R-Hs, and 3D R-Ls with varying relative density at a compressive speed of *V* = 5 m/s. As seen in Figure 17a, there is an increase in both initial peak stress and plastic energy dissipation with relative density for H-Hs. The results shown in Figure 17b,c suggest that the plastic energy dissipation and relative density have a positive correlation for re-entrant auxetics, which can be described as follows [37]:

(3) $$\sigma_{P}={\left(\frac{t}{L}\right)}^{2}\frac{\sigma_{Y}}{2\left(\cos\alpha -1\right)\cos\alpha }$$

where $\sigma_{P}$ is the plastic buckling stress, *t* is the wall thickness of regular hexagonal honeycomb, *L* is the side length of the unit cell, and $\sigma_{Y}$ is the yield stress of the structural material.

The increase in the structural relative density by increasing the wall thickness or beam cross-section area causes an increase in the plastic buckling stress, which leads to larger compressive stress and energy dissipation in the specified crushing distance of the structure.

#### 3.3.3. Effect of Unit Cell Number

**Figure 18:**
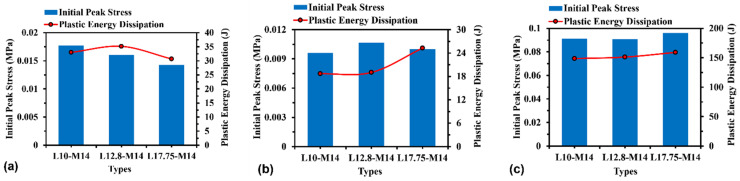
Initial peak force and plastic energy dissipation of (**a**) H-Hs, (**b**) R-Hs, and (**c**) 3D R-Ls corresponding to various unit cell numbers while the compressive speed remains constant at 5 m/s.

Figure 18 shows the effects of unit cell numbers on the initial peak stress and plastic energy dissipation of H-Hs, R-Hs, and 3D R-Ls. It can be noticed that in all cases, the unit cell number has a relatively slight effect on the initial peak stress and plastic energy dissipation due to the relatively good convergence for the structures with the given unit cell numbers in the present study.

#### 3.3.4. Effect of Material Property

**Figure 19:**
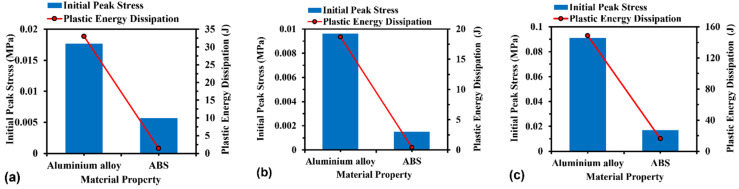
Initial peak force and plastic energy dissipation of (**a**) H-Hs, (**b**) R-Hs, and (**c**) 3D R-Ls corresponding to different material properties while the compressive speed remains constant at 5 m/s.

Figure 19 compares the initial peak stress and plastic energy dissipation of the structures with respect to the material properties of aluminium alloy and ABS, respectively. It is noticed that in all cases, the aluminium alloy models present higher initial peak stress and plastic energy dissipation than the ABS models due to the higher elastic modulus and yield stress. For instance, the initial peak stress of H-Hs, R-Hs, and 3D R-Ls increases by 3.1, 6.3, and 5.4 times, respectively, when changing the material type from ABS to aluminium alloy. The plastic energy dissipation of the ABS models for H-Hs, R-Hs, and 3D R-Ls rises by 23.9, 47.6, and 9.1 times, respectively, compared to that of the aluminium alloys.

## 4. Flexural Behaviour of Re-Entrant Auxetics

### 4.1. Failure Modes

**Figure 20:**
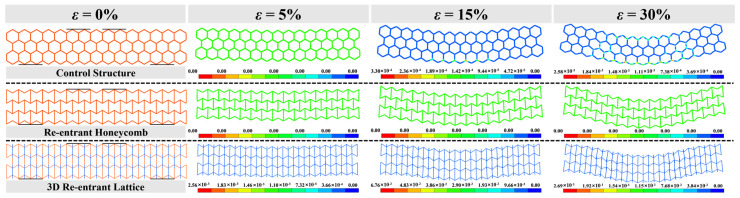
Deformation shapes with the flexural strain of H-H (**top**), R-H (**middle**), and 3D R-L (**bottom**).

Figure 20 presents the plastic strain field across the front face of the models. The plastic damage of 3D R-L initiates at 5% strain prior to that of H-H, which arises at 15% strain. No plastic behaviour can be observed for R-H over a total 30% strain, indicating the superiority of maintaining the elastic regime over a significant range of flexural strain. The plastic damage of H-H develops first in the tension zone at 15% strain and propagates to the compression zone at 30% strain. As for the flexural behaviour of 3D R-L, the plastic damage appears first in the compression zone and then propagates towards the tension zone. With the increase in the flexural strain, the 3D R-L exhibits dramatic shear deformation at 30% strain due to the high shear modulus.

### 4.2. Load–Deflection Curves

**Figure 21:**
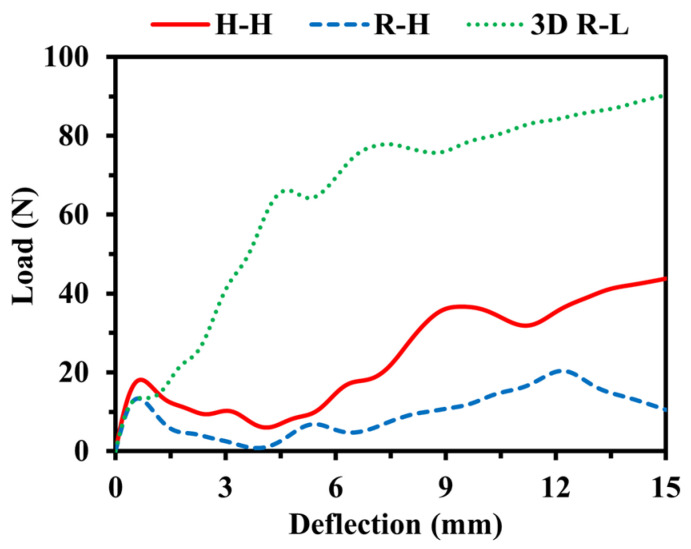
Impact load–deflection curves of various structures at an impact speed of *V* = 1 m/s while keeping the total mass constant at 0.028 kg.

In order to evaluate the dynamic performance of re-entrant auxetics under flexural loading, FE simulations of flexural tests were conducted at a test speed of *V* = 1.0 m/s where the total mass of the structure was kept constant. Figure 21 illustrates the load–deflection curves of the different models under flexural loading. The deflection here represents the moving distance of top rigid plates and reaches a maximum value of 15 mm, which corresponds to a flexural strain up to 30%. It can be found that compared to H-H and R-H, 3D R-L yields the maximum flexural load. Although R-H shows a small flexural load, it maintains the elastic deformation regime over a large range of strain. It should be mentioned that the load–deflection curves shown in the present study include the contribution from the backing plates.

#### 4.2.1. Effect of Relative Density

**Figure 22:**
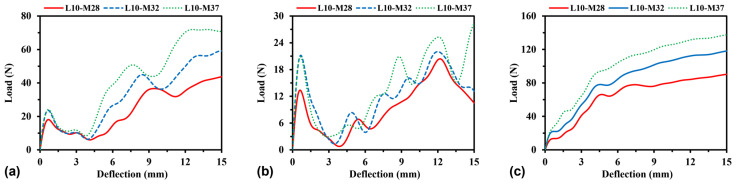
Load–deflection curves of (**a**) H-Hs, (**b**) R-Hs, and (**c**) 3D R-Ls with different values of relative density.

To evaluate the effect of relative density on the load–deflection relationship, the structural wall thickness for H-Hs and R-Hs and the cross-sectional area of beams for 3D R-Ls varied, while the side length *L* was kept constant. Accordingly, three values of the total model mass of 0.028 kg (M28), 0.032 kg (M32), and 0.037 kg (M37), respectively corresponding to the values of model relative density of approximately 0.016, 0.018, and 0.021, were considered for the parametric study. Figure 22 shows, respectively, the load–deflection curves corresponding to different values of model relative density for H-Hs, R-Hs, and 3D R-Ls. It can be observed that for all models, the flexural load increases with the increase in relative density. With the change in relative density from 0.016 to 0.021, the average flexural load is increased by 92%, 60%, and 53.3% for H-Hs, R-Hs, and 3D R-Ls, respectively. The flexural load of 3D R-Ls shows relatively lower sensitivity to structural relative density compared to that of the honeycomb structures, which is consistent with the previous finding under compressive load.

#### 4.2.2. Effect of Unit Cell Number

**Figure 23:**
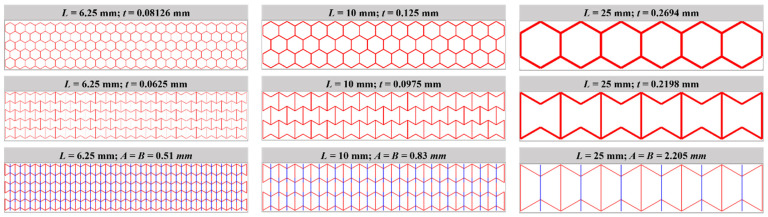
Geometry of flexural models when keeping total dimension and mass constant.

**Figure 24:**
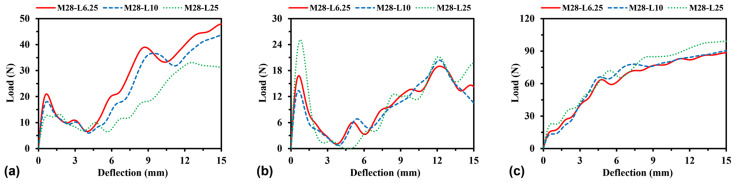
Load–deflection curves of (**a**) H-Hs, (**b**) R-Hs, and (**c**) 3D R-Ls corresponding to different unit cell numbers.

By changing the side length *L* between 6.25 mm and 25 mm, the unit cell numbers of the models changed accordingly from 24 × 5 to 6 × 1 (W × H), as shown in Figure 23. The effect of unit cell number on the load–deflection relationship is studied by keeping the total mass and dimension constant. Figure 24 displays the load–deflection curves for H-Hs, R-Hs, and 3D R-Ls corresponding to the different unit cell numbers. With regard to R-H and 3D R-L, the simulation results always remain convergent for the given unit cell numbers. However, as for H-H, reducing the unit cell number to 6 × 1 causes a dramatic reduction in the flexural load. These suggest that H-Hs need more unit cells than R-Hs and 3D R-Ls to eliminate the boundary effect under flexural load.

#### 4.2.3. Effect of Material Property

**Figure 25:**
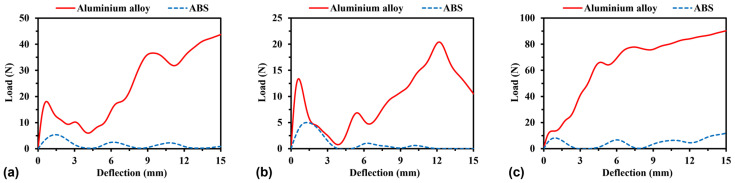
Load–deflection curves of (**a**) H-Hs, (**b**) R-Hs, and (**c**) 3D R-Ls corresponding to different material properties.

Figure 25 illustrates the load–deflection relationships of different types of models corresponding to the base materials of aluminium alloy and ABS. Compared to aluminium models, ABS models exhibit lower flexural load. It should be mentioned that the top rigid loading cells move more slowly than ABS models, resulting in a low flexural load. In this study, the replacement of ABS models with aluminium alloy models results in a rise in the average compressive stress of the H-Hs, R-Hs, and 3D R-Ls by 858%, 394%, and 2475%, respectively.

## 5. Conclusions

This work presents a numerical study to investigate the dynamic behaviour and energy dissipation performance of re-entrant auxetics, including 2D re-entrant honeycomb and 3D re-entrant lattice, in comparison with that of traditional hexagonal honeycomb, focusing on the effects of strain rate, relative density, unit cell number, and material property. Based on the simulation results, the main conclusions can be drawn as follows:

1. The strain rate plays a predominant role in the compressive failure mode and stress–strain curve characteristics of the different models. The principal failure mode for re-entrant auxetics under compressive loading is dynamic buckling at strain rates between 35.71 and 178.57 s^−1^. The material property could also influence the structural failure mode, while the relative density and unit cell number have a minor effect on the failure mode. The 2D re-entrant honeycomb shows a more significant negative Poisson’s ratio effect at high strain rates and is superior in maintaining the elastic deformation regime over a significant range of strain (e.g., 30%) to the hexagonal honeycomb and 3D re-entrant lattice under flexural loading.
2. Typically, the plastic energy dissipation of re-entrant auxetics under compressive loading exhibits a positive correlation with the strain rate and relative density, while the compressive and flexural behaviours of re-entrant auxetics are not dependent on the unit cell number. The 2D re-entrant honeycomb is more sensitive to material property. The initial peak stress and plastic energy dissipation rise by 6.3 and 47.6 times, respectively, when changing the material type from ABS to aluminium alloy.
3. The hexagonal honeycomb has greater capacity in impact resistance and plastic energy dissipation than the re-entrant honeycomb under low-speed (*V* = 5 m/s) compression, while the re-entrant honeycomb shows better performance than the hexagonal honeycomb in compressive resistance and plastic energy dissipation when subjected to high-speed (e.g., *V* = 25 m/s) compression. Under both low-speed and high-speed compressive loading, the 3D re-entrant lattice presents the optimum performance in plastic energy dissipation, but the maximum initial peak stress. Similar to the low-speed compression, the re-entrant honeycomb exhibits a small flexural modulus, but maintains the elastic deformation regime over a large strain range. This is an important characteristic for the application of re-entrant honeycomb as the core of composite structures for enhanced ductility under flexural loading.

## Figures and Tables

**Table 1:** Material properties used for FE simulations [12,25].

Material Properties	Specimens/Backing Plates (Aluminium Alloy 5052)	Specimens (ABS Polymer)	Rigid Plates/Rigid Supports/Rigid Loading Cells (Steel)
Young’s modulus, *E* (GPa)	70	2.2	210
Poisson’s ratio, *ν*	0.3	0.35	0.3
Yield strength, *σ_Y_* (MPa)	130	31	-
Density, *ρ* (g/cm^3^)	2.7	1.05	7.89

**Table 2:** Compressive failure modes for reference structures and re-entrant auxetics.

Type of Structure	Reference Structure			Re-Entrant Honeycomb			3D Re-Entrant Lattice		
	Compressive Velocity (m/s)			Compressive Velocity (m/s)			Compressive Velocity (m/s)		
	5	15	25	5	15	25	5	15	25
	FM	FM	FM	FM	FM	FM	FM	FM	FM
L10-M14	Q	D	D	T	D	D	D	D	D
L10-M16	Q	D	D	T	D	D	D	D	D
L10-M18	Q	D	D	T	D	D	D	D	D
L12.8-M14	Q	D	D	T	D	D	D	D	D
L17.75-M14	Q	D	D	T	D	D	D	D	D
L10-M14 (ABS)	G	D	D	D	D	D	T	D	D

Notes: L, M = side length, model mass; L10-M14 represents the simulated model has a side length of 10 mm and a model mass of 0.14 kg; Q = quasi-static failure modes (the major densification originates from the middle end of the structure), T = transitional failure modes (the major densification originates from both ends of the structure almost simultaneously), D = dynamic failure modes (the major densification originates from the proximal end of the structure), G = global failure modes [6].

## Data Availability

The data presented in this study are available on request from the corresponding author.
